# Composition Effects on the Morphology of PVA/Chitosan Electrospun Nanofibers

**DOI:** 10.3390/polym14224856

**Published:** 2022-11-11

**Authors:** Gustavo Cardoso da Mata, Maria Sirlene Morais, Wanderley Pereira de Oliveira, Mônica Lopes Aguiar

**Affiliations:** 1 Department of Chemical Engineering, Federal University of São Carlos, Rod. Washington Luiz, km 235, SP310, São Carlos 13565-905, SP, Brazil; 2 Faculty of Pharmaceutical Science of Ribeirão Preto, University of São Paulo, Av. do Café s/no, Bairro Monte Alegre, Ribeirão Preto 14040-903, SP, Brazil

**Keywords:** nanofibers, electrospinning, rheology, chitosan, PVA, membranes, response surface

## Abstract

Since the SARS-CoV-2 pandemic, the interest in applying nanofibers t air filtration and personal protective equipment has grown significantly. Due to their morphological and structural properties, nanofibers have potential applications for air filtration in masks and air filters. However, most nanofiber membrane materials used for these purposes are generally non-degradable materials, which can contribute to the disposal of plastic waste into the environment. Hence, this work aims to produce polyvinyl alcohol (PVA) and chitosan (CS) biodegradable nanofibers with controlled morphology and structure via electrospinning. An experimental design was used to investigate the effects of the PVA|CS ratio and concentration on the properties of the electrospinning compositions and electrospun nanofiber mat. The electrospinning parameters were constant for all experiments: Voltage of 20 kV, a feed rate of 0.5 mL·h^−1^, and a distance of 10 cm between the needle and a drum collector. CS proved to be an efficient adjuvant to the PVA’s electrospinning, obtaining a wide range of nanofiber diameters. Furthermore, 6.0% PVA and 1% CS were the best compositions after optimization with the response surface methodology, with a mean fiber diameter of 204 nm. The addition of biocide agents using the optimized condition was also investigated, using surfactants, citric acid, and pure and encapsulated essential oils of *Lippia sidoides*. Pure oil improved the material without enlarging the nanofiber sizes compared to the other additives. The nanofiber membranes produced have the potential to be used in air filtration or wound-dressing applications where biocidal activity is needed.

## 1. Introduction

Since the SARS-CoV-2 pandemic, the interest in applying nanofibers’ morphological and structural properties for air filtration in masks and air filters has increased. Several studies in the current literature reported the air filtration of bioaerosols using electrospun nanofibers [1,2,3,4,5]. Electrospinning is a versatile technique, generating micro and nanofibers with adequate properties for air filtration, such as a high surface-area-to-volume ratio, low basis weight, and uniform size [6]. Electrospun polyacrylonitrile (PAN) presented suitable properties [7,8], especially for air filtration [9]. Leung and Sun (2020) obtained efficient polyvinylidene fluoride (PVDF) nanofiber mats with fiber diameters between 84 and 524 nm for use in filtering a simulated SARS-CoV-2 virus [10,11]. However, these disposable materials, usually made with non-degradable polymers, are discarded into the environment, turning them into pollutants. Therefore, searching for highly efficient, low-cost, biodegradable air filtration materials for use in these types of personal protective equipment is an important research subject.

Recycled materials have also gained attention, especially for air filtration [12]. Plastics such as polyethylene terephthalate (PET) can be electrospun from raw PET bottles [13,14], producing materials with promising properties [15]. However, the solvents used to dissolve the polymers (e.g., trifluoracetic acid) are also a problem as they are usually aggressive, toxic, and difficult to handle. Therefore, using natural and biodegradable polymers and mild solvents can make electrospinning more environmentally friendly.

Polyvinyl alcohol (PVA) is a non-toxic and hygroscopic compound that has already been tested for air filtration [16]. It can reticulate its chains to confer resistance to moisture and other properties [17]. The addition of other materials can also confer biocide action, higher hydrophobicity, and improved mechanical properties [18,19,20,21,22]. The blend of PVA with low chitosan (CS) contents modifies the solution properties by improving its spinnability [23], resulting in hydrophobic fibers. The synthesis of membrane blends with higher contents of CS is possible via methods such as NIPS (nonsolvent-induced phase separation) [24] and freezing–thawing cycles [25]. Nevertheless, the electrospinning of chitosan is still problematic because of its high viscosity [26] and the need for electrical potentials of 4 kV·cm^−1^ or more [27]. Other additives, such as surfactants and essential oils (EOs), can also change the morphological properties of electrospun mats, aiming to improve the quality of fibers obtained and confer biocidal activity to the fibers.

Recently, PVA has been used as an excellent adjuvant for CS electrospinning [28] and is used in food packing [29], drug delivery [30], tissue regeneration [31], and wound dressing [32]. However, its harsh properties still hinder the process in areas such as air filtration. The main goal of this study is to produce a biodegradable material with high contents of CS without a loss of quality and control and optimize the nanofiber structure for further applications in air filtration with biocidal activity against pathogens such as SARS-CoV-2. The influence of rheological and solution properties was determined and linked to the properties of the formed electrospun nanofibers. Since PVA has no antimicrobial activity and chitosan’s activity depends on the pH and medium, adding a biocidal agent is necessary. Finally, certain additives such as surfactants, citric acid, and essential oil (pure and encapsulated) were investigated for the optimized PVA/CS electrospinning composition, evaluating the effects on the fiber quality and conferring biocidal activity to the material.

## 2. Materials and Methods

### 2.1. Materials

The polymers used in this study were polyvinyl alcohol (PVA) with a molecular weight of 85.500 g/mol and a degree of hydrolysis of 89.5% (Vetec Química Fina, Duque de Caxias/RJ, Brazil) and chitosan (CS) with a degree of deacetylation of 68.5% (Polymar, Rio de Janeiro/RJ, Brazil). Analytical-grade glacial acetic acid (G) at 99.0% (LabSynth, Diadema/SP, Brazil) was used as the solvent. The surfactant additives evaluated were sodium dodecyl sulfate at 95% (SDS—Neon, Suzano/SP, Brazil) and Cetyltrimethylammonium Bromide at 98% (CTAB, Sigma Aldrich, Saint Louis/MO, United States). The other additives used were citric acid (LabSynth, Diadema/SP, Brazil) and the essential oil of *Lippia Sidoides* (Produtos Naturais LTDA, Horizonte/CE, Brazil), pure and encapsulated into nanostructured lipid carriers (NLCs).

### 2.2. Electrospinning of the PVA|CS Compositions

The polymer solutions were separately prepared, with PVA varying from 6% to 12% (*w*/*v*) and CS from 1% to 4% (*w*/*v*), as described in Table 1. The polymers were weighed and dissolved in a water/glacial acetic acid system (30:70) maintained under magnetic stirring for three hours at a temperature of 80 to 90 °C for their complete dissolution. The samples of different concentrations of PVA|CS were then electrospun at varying operational parameters of the process to enhance the properties of the resulting fibers.

The electrospinning apparatus was composed of a high-voltage generator (0 to 50 kV) with a continuous current source (Electrotest HIPOT CC, Model EH6005C, Instrutemp, São Paulo/SP, Brazil), an infusion pump (Harvard Apparatus, Model Elite I/W PROGR SINGLE, Holliston/MA, USA), and a stainless-steel rotary cylinder (a diameter of 100 mm and a length of 200 mm) as the nanofiber collector. The electrospinning process occurred inside a fully grounded, electrically insulated compartment to minimize the occurrence of discharges during operation.

Preliminary tests evaluated the diameters of the syringe needles (0.55, 0.60, 0.70, and 1.20 mm) and the distance between the needle and collector (10, 11, and 12 cm). The data of the preliminary runs used to set the conditions are shown in the Supplementary Material (Table S1). A metallic needle with a 0.55 mm opening attached to a 5 mL plastic syringe fed the electrospinning formulations (Table 1). The electrospinning conditions were constant for all experimental runs: A flow rate of 0.5 mL·min^−1^, an electrical field of 20 kV, and a 3 h production duration. The metallic drum collector was covered with aluminum foil and placed at a distance of 10 cm from the needle tip, with the rotation speed set at 595 rpm. The electrospun nanofibers remained under ambient air for 1 h to volatilize the remaining acetic acid. The temperature and relative humidity in the electrospinning process was maintained at approximately 22 °C and 40%, respectively.

### 2.3. Morphology Characterization of the Electrospun Nanofibers

The morphology of the electrospun nanofibers was analyzed from photomicrographs obtained by scanning electron microscopy (SEM). First, 5 × 5 mm electrospun nanofiber mat samples were coated with carbon and gold in a Bal-Tec SCD Sputter Coater model-050 (Fürstentum/Liechtenstein) under a pressure of 0.1 mbar. The SEM photomicrographs were obtained with the model SEM FEI Inspect F50 (Eindhoven, The Netherlands), a FEG electron source with ETD and vCD detectors. The diameter distribution of the electrospun nanofibers was determined by image analysis from SEM micrographs using ImageJ^®^ software. Fourier-transform Infrared Spectroscopy (FTIR) was determined using a spectrophotometer FTIR Bomem MB-100 (ABB Bomem, Quebec, QC, Canada) with spectra ranging from 400 to 4000 cm.

### 2.4. Electrical Conductivity and Rheology of the Formulations

Determination of the electrical conductivity of the samples used a Metrohm 912 bench-top conductometer (Metrohm AG, Herisau, Switzerland) in triplicate measurements. To determine the rheology of each solution (duplicate assays), we used a Brookfield LV-DVIII coaxial cylinder Rheometer (Brookfield Engineering Laboratories Inc., Middleboro, USA) equipped with an SC4-18 spindle sensor. The spindle increased its rotation at a limited speed, then reversed the process, diminishing its velocity. Brookfield Rheocalc 3.2 software controlled the Rheometer and collected the experimental data on the shear rate and the corresponding shear stress.

### 2.5. Electrospinning of PVA|CS Compositions with Additives

The PVA|CS solution 6.0|1.00 (Table 1) was used to produce the samples loaded with additives at a concentration of 5% (*w*/*w*—dry basis). The new samples were kept under magnetic stirring for 30 min at 30 to 40 °C. The new samples were named according to their respective additive, namely, cetyltrimethylammonium bromide (6.0|1.00/CTAB), citric acid (6.0|1.00/Cit), and sodium dodecyl sulfate (6.0|1.00/SDS).

Essential oil (EO) was extracted from *Lippia sidoides*—popularly known as pepper-rosemary—an aromatic Brazilian shrub with proven antimicrobial activity [33]. Three samples with *Lippia sidoides* EO were used, namely, pure oil (6.0|1.00/EO) and the EO loaded in nanostructured lipid carriers (6.0|1.00/NLC-Com and 6.0|1.00/NLC-BC) [34]. The NLCs’ constituents were the essential oil of *Lippia sidoides*, SDS, oleic acid (Vinhedo/SP, Brazil), and a solid lipid. The solid lipid for the sample NLC-Com was Compritol^®^ 888 ATO (Gattefossé, Saint-Priest, France), while the sample NLC-BC used a mixture of beeswax (Via Farma, São Paulo/SP, Brazil) and carnauba wax (Foncepi, Fortaleza/CE, Brazil). A detailed description of the NLC-Com (F8) and NLC-BC (F18) preparations was reported by Baldim and coworkers (2022) [35].

## 3. Results and Discussion

### 3.1. Morphological Structure of the Fibers

**Figure 1:**
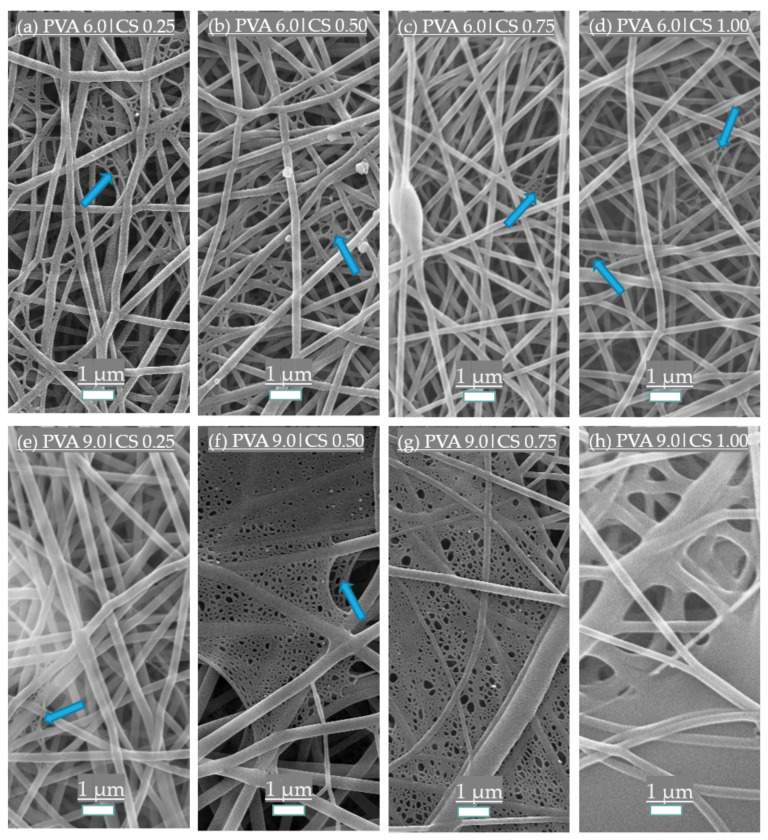
SEM photomicrographs from samples with fixed PVA series at 6.0%: (**a**) CS 0.25; (**b**) CS 0.50; (**c**) CS 0.75; and (**d**) CS 1.00%. A series with 9.0% PVA with (**e**) CS 0.25; (**f**) CS 0.50; (**g**) CS 0.75; and (**h**) CS 1.00% is also presented. Arrows indicate the spider nets.

To observe the real influence of the solutions’ concentrations of PVA and CS on the fibers’ properties, selected samples shown in Table 1 were electrospun and submitted to an SEM analysis (Figure 1). PVA has been shown to be effective as an adjuvant for the electrospun CS polymer, forming smooth fibers without beads. Chitosan solutions below 2% do not have sufficient material to form a fibrous structure, while solutions above 2% are too viscous for electrospinning [36]. The high viscosity occurs due to strong hydrogen bonds between the NH_2_ and OH groups of the CS chains. Adding PVA seems to diminish the degree of those interactions between the CS chains due to interactions between the two polymers’ chains. Since Newtonian fluids such as PVA are not supposed to entangle, those interactions diminish the CS chains’ entanglement threshold, making spinnability possible. The same effect was observed by reducing the molecular weight of CS and, as a consequence, its entanglement degree [26]. Other alternatives previously investigated include using cosolvents such as DMSO to reduce entanglement and improve the chitosan’s spinnability [36].

PVA addition also creates structures of different degrees in the nanofiber mat, known as spider-net webs [6,37]. PVA 6.0% shows a sparse and well-distributed spider-net structure alongside the fiber mat that appears denser at higher contents (PVA 7.5 and 9.0%) with smaller pores. At these high contents of PVA, fiber fusion, film layers, and aggregates appear more often. The formation of the spider-net structures can improve the PVA’s nanofibers mat strength, increasing the network resistance to deformation [38]. However, in air filtration applications, a dense spider-net web close to forming a film may significantly increase the pressure drop to unacceptable values, impeding its use.

#### Fourier-Transform Infrared Spectroscopy (FTIR)

**Figure 2:**
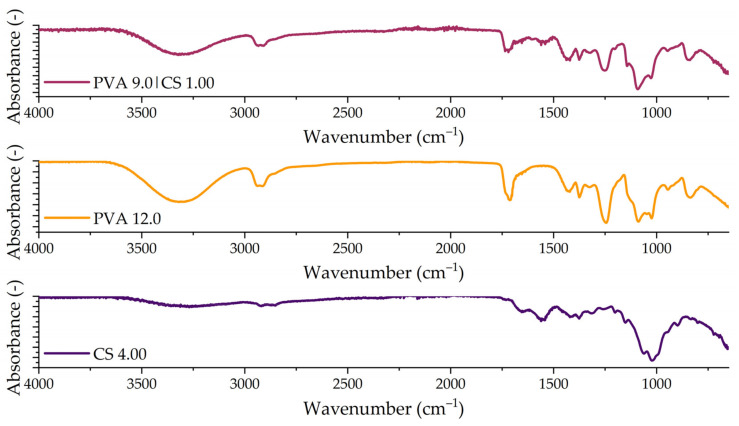
FTIR spectra of the pure samples (PVA 12.0 and CS 4.00) and after the blend at the PVACS ratio of 75:25 (PVA 9.0|CS 1.00).

Figure 2 shows FTIR results obtained for the pure solutions of PVA at 12.0% and CS at 4.00% and their mixtures—sample PVA 9.0|CS 1.00. The occurrence of certain interactions between the PVA and CS functional groups can be seen in Figure 2. The characteristic PVA groups, CH_2_ and C–O, are represented by bands at 2923 cm^−1^ and 1082, respectively [31]. The stretching of the OH group causes a peak at 3305 cm^−1^, and the 835 cm^−1^ band is related to the C–C resonance group [39].

The band at 1154 cm^−1^ represents the resonance of the chitosan saccharide structure. The band at 1074 cm^−1^ is related to the stretching vibrations of C–O–C glycosidic linkages [31]. Peaks at 1074 and 1380 cm^−1^ represent the amide C–N bending vibrations and the C–H amide group [39]. The absorption band in the range of 1650 to 1655 cm^−1^ is related to vibrational C=O, caused by partial deacetylation of chitin and characteristic of the secondary amide groups [40]. The broad band between 3000 and 3600 cm^−1^ is related to the N–H stretching of primary amino groups [41], while the band at 1575 cm^−1^ is associated with N–H bending [42]. The band at 896 cm^−1^ is evidence of Chitosan OH out-of-plane ring stretching [28].

The out-of-plane OH vibration band disappears after the blending of PVA and CS. The formation of hydrogen bonds between PVA and CS molecules [28] is associated with the broadening of the absorption band between 3000 and 3600 cm^−1^. Stretching vibrations diminish with lower frequencies and hydrogen bonds, leading to intermolecular bonds between the CH_2_-OH group in CS and the hydroxyl groups of PVA [31,41]. Çay and colleagues [2014] reported the disappearance of the PVA band 1260 cm^−1^ after blending with CS. In our study, the band remains after the blend, showing O-H structures [41]. The peak at 1708 cm^−1^ is associated with the carbonyl of polyvinyl acetate, showing other possible bonding structures [43].

### 3.2. Statistical Analysis and Fiber Optimization

#### 3.2.1. Multilevel Factorial Design with Response Surface Analysis

**Figure 3:**
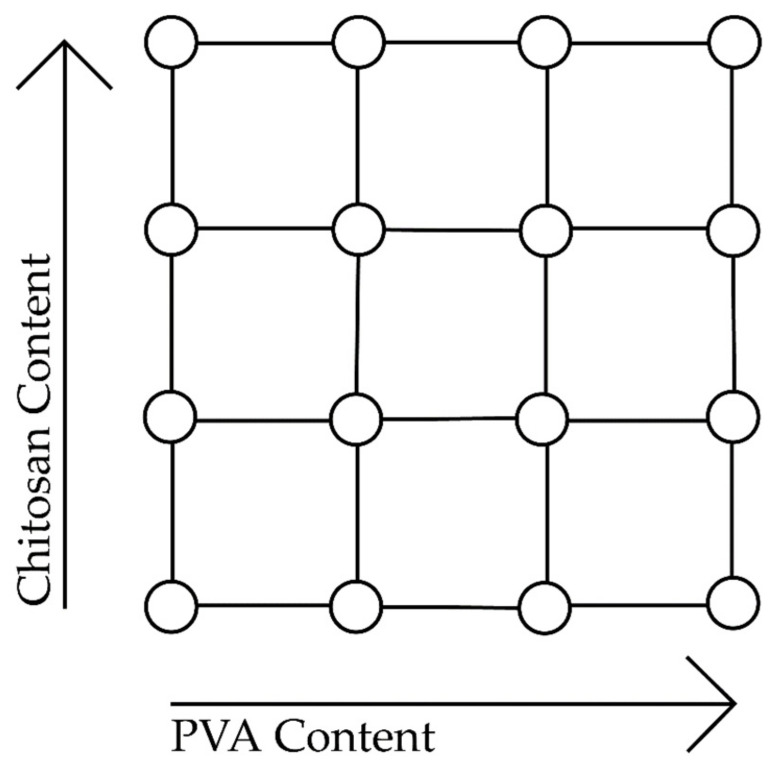
Model of a 4^2^ factorial design scheme.

This study used a 4^2^-factorial design, using Minitab^®^ to evaluate the composition effects (Table 1) on the formulation properties (rheology consistency index (K), flow index (*n*), and the solution conductivity (σ)), and nanofiber diameters (nm). The regression equation used to generate model $\hat{y}$ follows the structure of a polynomial sequence, with $x_{i}$ representing the factors, $x_{i}^{2}$ representing the quadratic terms of the binary interaction between each factor (i.e., interaction with itself), and $x_{i}x_{j}$ represents the interaction between the distinct factors. Equation (1) shows the regression model applied.

(1) $$\hat{y}=\beta_{0}+\beta_{1}x_{1}+\beta_{2}x_{2}+\beta_{11}x_{1}^{2}+\beta_{22}x_{2}^{2}+\beta_{12}x_{1}x_{2}$$

where $\hat{y}$ represents the variable analyzed, while $\beta_{ij}$ is the constant coefficient for each term. Figure 3 exemplifies a scheme of the model.

**Figure 4:**
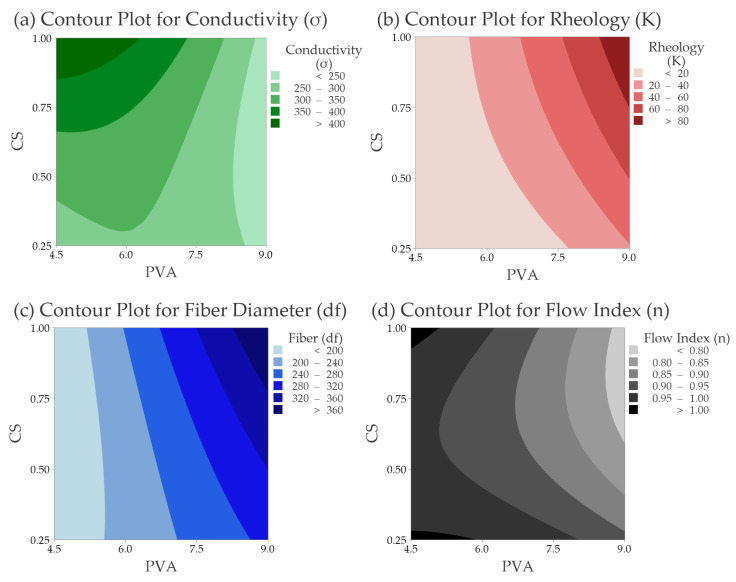
Contour plots generated by regression models for each experimental response: (**a**) Electrical conductivity; (**b**) rheology consistency index (K); (**c**) fiber diameter; and (**d**) flow index.

The linear terms (PVA and CS) and the interaction term (PVA × CS) significantly influence the electrical conductivity and rheology consistency index responses, which are consistent with the polymer interchain interactions hypothesis. Only the PVA factor is significant in the fiber Diameter (df) response and the Flow Index (*n*). This result does not necessarily mean that the CS does not influence those properties, but its effect was not high enough to be detected in the statistical analysis. Figure 4 shows the contour plots resulting from the regression analysis.

The regression models took into account the linear properties’ terms (PVA and CS), square terms (PVA × PVA and CS × CS), and interaction terms (PVA × CS). Table 2 shows the regression analysis with the level of statistical significance (*p*-value). As can be observed, the models fitted for conductivity (σ), the rheology consistency index (K), and fiber diameter (df—nm) are highly significant. In contrast, the flow index (*n*) model does not show statistical significance.

#### 3.2.2. Electrical Conductivity (σ)

Table 3 presents the electrical conductivity of the pure CS solutions and PVA|CS mixtures. The influence of PVA reduces the electrical conductivity of the solutions, as seen in the main effects and interaction plots ((Figure S1a,b) of the Supplementary Material). For the 9.0% PVA series, the resulting conductivity is inferior to the pure solution of PVA. The high PVA content may create more hydrogen bonds with the CS, diminishing the chemical groups responsible for electrical conductivity. The molecular structure of PVA in a medium with an excess of H^+^ ions (acid solution) inhibits the protons’ dissociation, diminishing the electrical conductivity of the solutions. Higher electrical conductivity may be beneficial for the electrospinning process. However, too-high electrical conductivity can prejudice fiber formation, leading to the formation of aggregates films, as usually seen in the electrospinning of chitosan solutions [27]. Values between 400 and 450 μS·cm^−1^ appear ideal for the conductivity of PVA|CS solutions. As seen in the contour plots (Figure 4a,c), an increase in electrical conductivity leads to a decrease in the nanofiber diameter for the level ranges studied. Some authors reported similar results [37,44].

#### 3.2.3. Rheology Consistency Index (K) & Flow Index (*n*)

The solutions of the polymers used in electrospinning are usually Newtonian fluids or pseudoplastic. The *Ostwald* de Waele power law model, described in Equation (2)**,** is typically applied to describe the pseudoplastic (shear thinning) behavior of fluids:

(2) $$\tau =K\cdot \gamma^{n}$$

where τ is the shear stress (or applied force/area, Dyn·cm^−2^), *γ* is the shear rate (or resulting deformation, 1.s^−1^), and n (flow index, dimensionless) K (consistency index, Dyn·cm^−2^·s) are the constants [45,46]. OriginLab^®^ was used to process the rheological and morphological data. The Rheology Consistency Index (K) is the Power Law model’s angular coefficient and is responsible for controlling the proportion of shear stress per shear rate and, consequently, the viscosity. The flow index (*n*) is the exponential fraction of the power law representing its curvature. The rheology consistency index (K) is closely related to the nanofiber size as the mean fiber diameter increases with the K value [47]. Surface tension dominates the electrospun jet by reducing the ratio of polymer chains per solvent, resulting in thinner fibers [48], as shown in Figure 4.

Pseudoplastics fluids can also display time-dependent behavior [49]. Thixotropic solutions show a high initial deformation resistance, further relaxing as the applied shear force increases, while rheopetic systems increase their resistance and viscosity with stress over time. The shift from rheopexy to thixotropy of the solutions with PVA increment is a factor relevant to the process. Thixotropic polymer solutions present a high initial deformation resistance. Therefore, a higher electrical field is needed to overcome the initial resistance (e.g., pure CS 1.00% is electrospun only at 40 kV [27]).

The fiber formation depends on the polymer solution concentration and kinematic viscosity [50]. When an electrical field is applied to a polymer solution pendant droplet, it can assume the stable shape known as the Taylor cone [51]. This results from the equilibrium forces between the surface tension and the electrical charges on the droplet. Depending on the nature of the polymer solution (e.g., Newtonian, inviscid, or viscoelastic), it can assume different shapes [45]. The rheopetic behavior appears to favor fiber formations of smaller sizes since the rheopetic solutions presented a smaller mean fiber diameter. The low initial resistance allows the electric forces to deform the Taylor Cone more easily, facilitating the elongation step. During elongation, fibers gain resistance (by increasing the viscosity), avoiding breaks and thinning the fibers.

**Figure 5:**
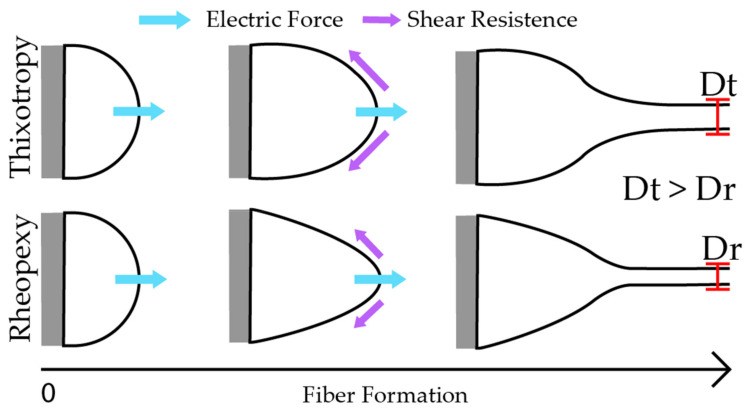
Taylor cone deformation according to the rheology properties. Thixotropic behavior leads to thicker fibers when compared with rheopetic behavior.

**Figure 6:**
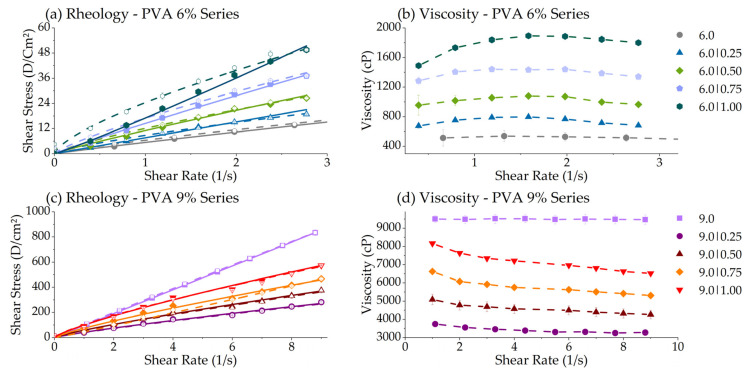
Rheograms for the PVA fixed series of 6.0% analyzed on the range of 0 to 3 s^−1^: (**a**) Rheology and (**b**) viscosity. Rheograms for the PVA fixed series of 9.0% analyzed in a range of 0 to 9 s^−1^: (**c**) Rheology and (**d**) viscosity. Close dots and solid lines show the increase in shear stress, while open dots and dash lines show tensile stress relaxation.

In contrast, during the Taylor cone phase, the initial resistance of the thixotropic behavior hinders the next stage of fiber formation, the elongation step. Similarly, the deformation resistance of thixotropic solutions diminishes in the elongation step (i.e., a decrease in viscosity), making it easier for jets to break. Fibers with high flexibility are prone to forming better network structures due to interfiber interactions [38]. Figure 5 exemplifies the process. Figure 6 shows the rheograms for certain sample series studied.

The 6.0% PVA series presented rheopetic behavior (flow index higher than 1.0), increasing this characteristic alongside the CS content. The viscosity follows the same trend, growing with the shear rate. The PVA 9% series shows the opposite direction, decreasing its viscosity over time and exhibiting thixotropic behavior (a flow index lower than 1.0). The surface tension dominates the electrospun jet by reducing the ratio of polymer chains per solvent molecule, resulting in thinner fibers [48]. Adding CS to the pure PVA at 6.0% increased its pseudoplastic behavior and solution viscosity. For pure PVA at 9.0%, however, adding CS produced the opposite effect, diminishing the viscosity. They are so viscous that the fibers show difficulties in flowing and elongating. These compositions also presented poor spinnability. The morphology results showed that the too-high PVA viscosities at 9.0% produced large fibers.

For PVA|CS solutions, K values under 30.0 Dyn·cm^−2^·s lead to thinner fibers. Table S2 presents the values of K (consistency index) and *n* (flow index) for all compositions with regression plot data. The rheology consistency index is closely related to nanofiber size since the mean fiber diameter increases with the K value [47].

#### 3.2.4. Fiber Size Diameter (df)

**Figure 7:**
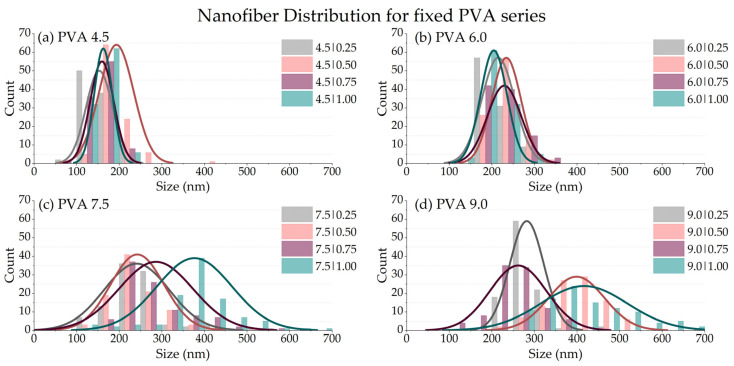

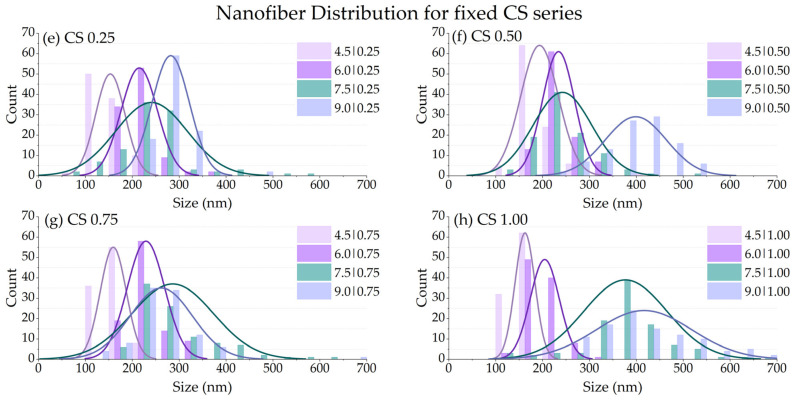
Fiber size distribution for samples with fixed PVA content series: (**a**) 4.5; (**b**) 6.0; (**c**) 7.5, and (**d**) 9.0% *w*/*w*. Fiber size distribution for the series with fixed CS at: (**e**) 0.25; (**f**) 0.50; (**g**) 0.75, and (**h**) CS 1.00%.

The fiber mat originating from the PVA 6.0|CS 1.00 solution was chosen because of the high content of chitosan and good spinnability. The minor presence of spider-net webs guarantees structural resistance without blocking the mat pores, as seen in the other PVA solutions with high CS contents. The rheological properties of PVA 6.0|CS 1.00 and the pure CS 1.00 solution were almost identical. Therefore, adding PVA at this concentration does not change the solution’s rheology significantly, causing a high decrease in the formulation’s electrical conductivity (from 1505.9 for Pure CS 1.00 to 393.3 μS·cm^−1^ for PVA 6.0|CS 1.00). The fiber size distribution was determined for the different series according to PVA and CS contents, as shown in Figure 7.

For series with a low fixed PVA content (4.5 and 6.0), downward variation occurs with the increase in CS, while a wider distribution is evidenced at higher contents (7.5 and 9.0). For the fixed series of CS, the increase in PVA dislocates the distributions for the samples with low PVA (4.5 and 6.0). Table 4 shows the average fiber diameter for each combination of PVA|CS blends. Table S3 shows the quality of the produced fibers.

The high proportion of PVA relative to CS overcomes the chitosan’s influence on the final fiber dimensions. Fiber sizes smaller than 250 nm in diameter are beneficial for the air filtration of viruses and small nanoparticles by improving the collection mechanisms of nanoparticles [15,52,53]. Smaller fibers can create a too-sinuous passage, increasing the pressure drop.

Other parameters can also interfere with fiber formation. The rise in flow rate squeezes more solution through the needle, increasing the fiber diameter [50]. With more material on the pendant droplet, slight changes in the surface charge density can influence the fiber’s evolution [54]. Water in the atmosphere can also affect the formation of the Taylor cone structure. Depending on the relative humidity, the fibers tend to absorb the water from the air during the elongation step. Water condensation releases latent heat. In response, the fibers expel the solvent earlier, preventing fiber solidification [55]. Water retention between polymer chains may be responsible for agglomerate formation and fiber fusion [37]. PVA hygroscopicity increases the fibers’ water affinity, especially in samples with content as high as PVA 9.0%. The shift in the binary solution of water-acetic acid also changes the solubility of the jet [55,56]. As chitosan is hydrophobic, the excess water is a driving force for CS agglomeration by separating the phases [56,57].

#### 3.2.5. Validation of the Response Surface Analysis

**Figure 8:**
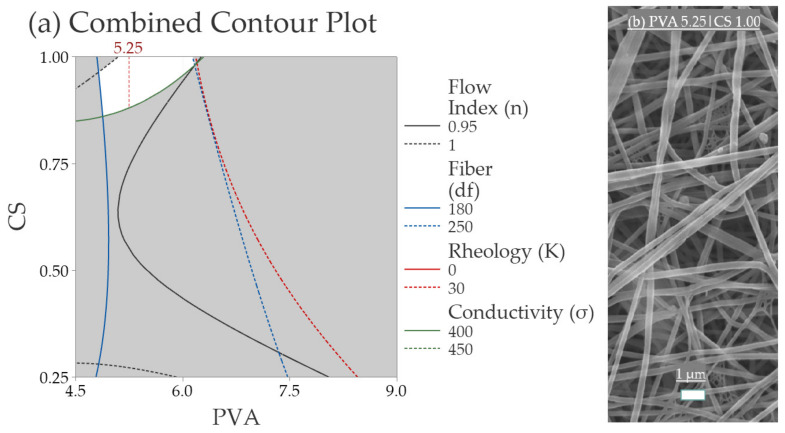
(**a**) Combined contour plots of the electrical conductivity, rheology consistency index, fiber diameter, and flow index, with defined margins for its properties. The white region shows the position of the validation assay; (**b**) SEM image of the sample with PVA 5.25%|CS 1.00%.

This study aimed to find the optimal region inside the generated model. A combination of the contour plots for all responses was constructed and is shown in Figure 8.

The blank area in the graph represents the optimal region for all responses. To validate the model, a sample containing PVA 5.25|CS 1.00 was prepared and electrospun. The resulting properties for the sample were expected to be inside the white region of Figure 8a. The experimental mean fiber diameter of the test sample was 222.0 ± 36.4 nm inside the white area. The spider-net web was also present but more significant than those observed in the optimal point (6.0|1.00). It was observed that by diminishing the PVA content from 6.0, the presence of spider-net increases.

### 3.3. Additives Influence

The addition of nanoparticles [4], surfactants [58], curcumin [21,59], and essential oils [60] has already been used to improve electrospun fibers’ properties. Adding antimicrobial essential oils, such as the *L. sidoides* EO or its encapsulated forms, has proved to be a viable option for fiber functionalization, producing fiber mats with biocidal activity.

#### 3.3.1. Surfactant Addition

**Figure 9:**
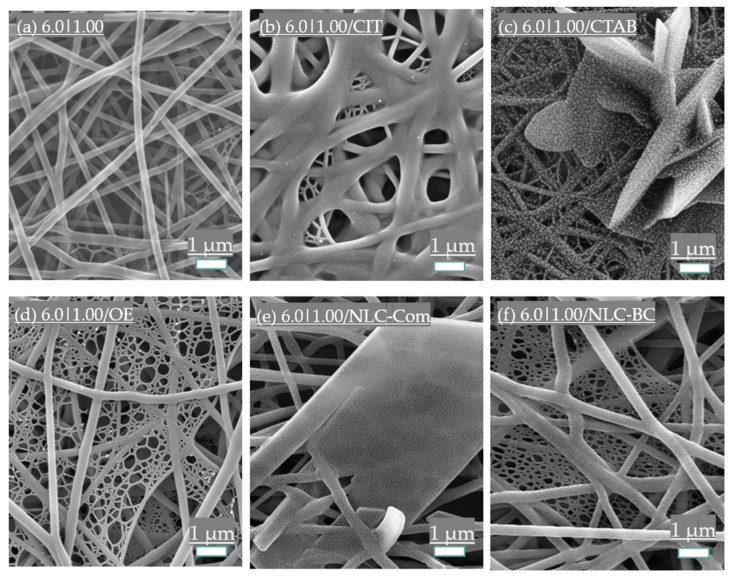
SEM images of the 6.0|1.00 solution (**a**) without additives and with 5% additions of (**b**) citric acid, (**c**) cetyltrimethylammonium bromide (CTAB), (**d**) essential oil of *Lippia sidoides* and *L. sidoides* loaded in nanostructured lipid carriers (NLC) used as solid lipids, (**e**) Compritol^®^ 888 ATO, and (**f**) blend of beeswax and carnauba wax.

Surfactants are known for their ability to reduce the surface tension of solutions, favoring the electrospinning process [61]. Surface tension also has an essential effect on the morphologies of natural polymers’ electrospun fibers, influenced by the solution’s components [62]. Figure 9 shows the SEM analysis of the electrospun fibers with the additives.

Surprisingly, the surfactant samples (6.0|1.0/CTAB and 6.0|1.0/SDS) did not show the same trend. Figure 9c shows that the samples of PVA and CS with CTAB formed crystal structures that are likely composed of CTAB. Barakat and colleagues (2009) observed similar behavior in solutions containing NaCl [37]. The presence of CTAB also appears to prejudice the gold deposition and coating of the electrospun fibers for SEM analysis. As previously reported, citric acid appears to create a crosslink between the electrospun fibers [17,63]. The crosslink improves the material resistance and reduces the hydrophobicity but prejudices the air passage.

**Figure 10:**
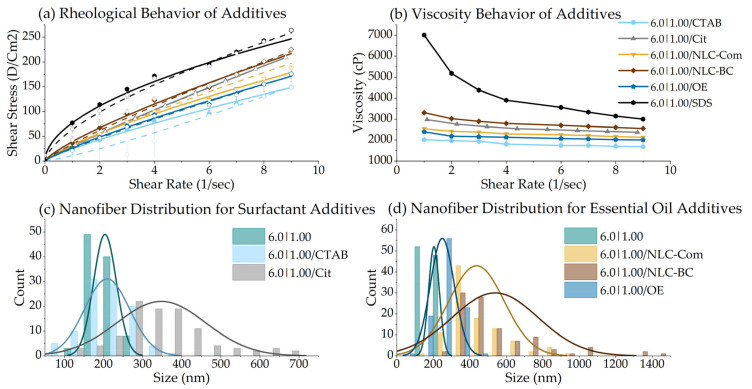
(**a**) Rheograms for the PVA 6.0|CS 1.00 solution with additives, analyzed at 0 to 10 s^−1^. Close dots and solid lines show the increase in shear stress, while open dots and dash lines show tensile stress relaxation. (**b**) Viscosity behavior of the samples under increasing shear stress; (**c**) fiber size distribution for the samples with surfactants as additives; and (**d**) fiber size distribution for the samples with essential oil and NLCs as additives.

The effects of the additives on compositions’ rheology and nanofiber size distributions are shown in Figure 10. The sample with SDS (6.0|1.0/SDS) was not spinnable. As shown in Figure 5b, the high initial viscosity and the rheology of the solution with SDS become a hindrance, favoring an electrospraying process. Viscoelastic force and interfacial surface tension significantly influence electrospraying [64]. Since surfactants such as Triton X-100 and SDS have already been proven beneficial for PVA fiber formation [17], this solvent may be the answer to this distinct behavior. Yang and coworkers (2004) [65] tested different solvents with electrospun polyvinyl pyrrolidone fibers. They reduced the surface tension of the solution at constant concentrations and observed that bead formation diminished. Adjusting the solvent’s ratio caused changes in the viscosity and surface tension [65]. The pH may also influence surfactant adsorption. pH can affect the number of hydroxyl groups on the surface, affecting the formation of hydrogen bonding [66]. The surfactants and citric acid also changed the entanglement of molecules and the interchain interaction, diminishing the spinnability, perhaps by changing the surface tension. The pH of the sample solutions described in Table 1 is presented as Supplementary Data (Table S4).

#### 3.3.2. Essential Oil and Nanostructured Lipid Carriers (NLCs) Incorporation

NLCs are blends of solid and liquid lipids with a surfactant to reduce the interfacial tension between the NLC and a continuous aqueous phase. NLC presents advantages over polymeric and inorganic particles, as it is biodegradable and presents reduced toxicity. The high load capacity and its chemical versatility increase their range of applications [34]. This study used oleic acid as a liquid lipid and SDS as a surfactant carrier for the *Lippia sidoides* essential oil. Compritol^®^ 888 ATO was used for the composition of 6.0|1.00/NLC-Com as a solid lipid, while beeswax and carnauba wax were used for the 6.0|1.00/NLC-BC composition.

Pure *Lippia sidoides* essential oil (6.0|1.0/EO) positively affects fiber formation. This sample slightly increases the mean fiber size and induces the massive formation of spider-net structures (subject of future work). However, the spider nets were opened with large pores instead of a closed membrane, as observed in other samples with those structures. This result may be a positive aspect for applying the electrospun fibers in air filtration.

Since the main constituent of the essential oil of *Lippia sidoides* is composed of thymol (~68%) [33,35], this component may have primary responsibility. Thymol was already used in electrospinning to produce non-toxic and biocide polyurethane skin-like membranes [67] and hydrophobic porous fibers of cellulose acetate [68], among others [69,70,71]. It is a proven biocidal agent against viruses [72], bacteria [73,74], and fungi [33,75] and is a promising agent against SARS-CoV-2 [76]. Our research group has ongoing studies evaluating the virucidal activity of *Lippia sidoides* compositions against SARS-CoV-2.

The NLCs increased the fiber sizes. Possible NLC disruption during the electrospinning process may release lipids amid the solution. As beeswax and carnauba wax have more complex structures than Compritol^®^ 888 ATO, it is natural that 6.0|1.00/NLC-BC promotes an increase in fiber sizes compared with 6.0|1.00/NLC-Com. The presence of SDS in their compositions may also be relevant since the samples with the addition of SDS did not produce fibers.

## 4. Conclusions

The variation in the PVA and CS solutions’ compositions significantly influenced the resulting fiber mats, changing the nanofibers’ structure and size. It is possible to predict and control the final fiber diameter, a relevant factor for air filtration purposes, by optimizing the distinct composition properties, such as the rheological behavior and the electrical conductivity.

The optimal composition was set at 6% PVA and 1% CS, since a higher content of CS tended to obstruct the fiber mats’ pores. Fiber with a mean size of 204.4 ± 32.2 nm was obtained for this specific composition using a voltage of 20 kV, a flow rate of 0.5 mL·h^−1,^ and a 10 cm distance between the needle and the drum collector. The fiber mat did not present beads but formed a spider-net web structure that can be beneficial or prejudicial to air filtration. Hence, the presence of spider-net webs in air filtration deserves further investigation. The biodegradable fiber mats loaded with additives may be suitable for air filtration or wound-dressing applications where biocidal action is needed.

## Figures and Tables

**Table 1:** Composition of the blended PVA and CS (PVA|CS) formulations. The PVA and CS contents show the concentration of the previous solution. The resulting values show the concentration of polyvinyl alcohol relative to chitosan (PVA|CS) after mixing in the proportion of 75:25.

Chitosan (CS) Content	Polyvinyl Alcohol (PVA) Content			
	6%	8%	10%	12%
1%	4.5|0.25	6.0|0.25	7.5|0.25	9.0|0.25
2%	4.5|0.50	6.0|0.50	7.5|0.50	9.0|0.50
3%	4.5|0.75	6.0|0.75	7.5|0.75	9.0|0.75
4%	4.5|1.00	6.0|1.00	7.5|1.00	9.0|1.00

**Table 2:** Models fitted to experimental data of electrical conductivity (σ), consistency index (K), fiber size (nm), and flow index (*n*) and its statistical significance (*p*-value).

Response	Model	*p*-Value
σ (mS·cm^−1^)	−73 + 109.0 PVA + 266 CS − 7.90 PVA × PVA + 150 CS × CS − 50.4 PVA × CS	0.001
K (Dyn·cm^−2^)	57.5 − 22.3 PVA − 53.5 CS + 1.951 PVA × PVA − 15.5 CS × CS + 16.92 PVA × CS	0.000
df (nm)	108 + 17.5 PVA − 224 CS − 0.02 PVA × PVA + 46 CS × CS + 34.7 PVA × CS	0.005
*n*	0.949 + 0.055 PVA − 0.268 CS − 0.00487 PVA × PVA + 0.381 CS × CS − 0.0429 PVA × CS	0.128

**Table 3:** Electrical conductivity (μS·cm^−1^) of pure CS solutions and CS/PVA mixtures (% *w*/*w*).

Chitosan Content	Polyvinyl Alcohol Content				
	0.0	4.5	6.0	7.5	9.0
0.00	-	238.1 ± 6.9	231.9 ± 6.8	249.7 ± 15.6	286.4 ± 30.4
0.25	519.9 ± 7.0	312.9 ± 17.8	254.3 ± 2.6	247.6 ± 7.9	278.8 ± 6.1
0.50	826.5 ± 6.7	326.9 ± 6.2	301.8 ± 10.4	300.8 ± 1.7	180.5 ± 12.5
0.75	1176.3 ± 8.2	347.9 ± 7.2	363.2 ± 4.1	350.9 ± 8.6	195.3 ± 11.9
1.00	1505.9 ± 14.5	445.9 ± 4.5	396.2 ± 5.6	369.7 ± 1.9	211.8 ± 8.6

**Table 4:** Average fiber diameter (nm) for the electrospun fibers obtained from different solutions of PVA and CS (PVA|CS).

Chitosan (CS) Content (% *w*/*w*)	Polyvinyl Alcohol (PVA) Content (% *w*/*w*)			
	4.5	6.0	7.5	9.0
0.25	153.1 ± 31.2	214.8 ± 38.7	240.9 ± 76.8	282.4 ± 40.2
0.50	193.4 ± 40.5	234.0 ± 34.4	242.3 ± 62.6	398.8 ± 65.5
0.75	159.3 ± 27.8	229.1 ± 39.9	286.2 ± 87.1	261.8 ± 66.6
1.00	162.4 ± 21.2	204.4 ± 32.2	376.7 ± 89.0	416.4 ± 101.8

## Data Availability

The data presented in this study are available on request from the corresponding author.

## Supplementary Materials

The following supporting information can be downloaded at: https://www.mdpi.com/article/10.3390/polym14224856/s1, Table S1: Parameter data of nanofibers produced by electrospinning in this study. Table S2: Data obtained by the rheological analysis of sample solutions described in Table 1. Table S3: Qualitative analysis of fibers’ electrospinnability of compositions in Table 1. Table S4: pH of the sample solutions described in Table 1. Figure S1: Main effects and the resulting interaction plot for each variable, respectively: (a,b) Conductivity; (c,d) rheology consistency index; (e,f) fiber diameter; and (g,h) flow index.
