# Supplementary material for: Composition Effects on the Morphology of PVA/Chitosan Electrospun Nanofibers
Source: Polymers (Basel). 2022 Nov 11;14(22):4856. doi: 10.3390/polym14224856 (PMC9698655; doi:10.3390/polym14224856)
Supplement: Supplementary file 1 [file polymers-14-04856-s001.zip › polymers-1975555-supplementary.pdf]

Supplementary Materials

Composition Effects on the Morphology of PVA/Chitosan Electrospun Nanofibers

Gustavo C. Mata[1, †], Maria Sirlene Morais[2, †], Wanderley Pereira Oliveira[2], Mônica Lopes Aguiar[1, *]

Table S1: Parameters data of the nanofibers produced by electrospinning in this study.

Sample	PVA (% w/w)	Chitosan (% w/w)	Solvent	Production Time (h)	Proportion (PVA:CS)	Needle Inner Diameter (mm)	Temperature (°C)/ Relative Humidity (%)
P1	7	4	Water	-	75:25	-	-
P2	7	4	Water	-	50:50	-	-
P3	7	4	Water	-	25:75	-	-
P4	12	4	Water	-	75:25	-	-
P5	12	4	Water	-	50:50	-	-
P6	12	4	Water	-	25:75	-	-
0	7	4	AcOH	3	75:25	0.3	-
1	7	4	Water	2	75:25	0.6	-
2	7	4	AcOH	2	75:25	0.6	-
3	7	4	AcOH	2	75:25	0.6	-
4	7	4	Water	2	75:25	0.6	-
5	7	4	Water	2	75:25	0.6	-
6	12	4	Water	2	75:25	0.6	-
7	12	4	Water	2	75:25	0.6	-
8	7	4	AcOH	2	75:25	0.6	-
9	7	1	AcOH	2	75:25	0.6	-
10	12	1	Water	2	75:25	0.6	-
11	7	1	AcOH	2	75:25	0.6	-
12	7	1	AcOH	2	75:25	1.2	-
13	12	1	AcOH	3	75:25	0.6	-
14	7	2	AcOH	2	75:25	0.7	-
A	7	4	Water	3	75:25	0.7	-
B	12	1	Water	3	75:25	0.7	-
9.0|0.25	12	1	AcOH	3	75:25	0.55	-
9.0|0.50	12	2	AcOH	3	75:25	0.55	26.3/34
9.0|0.75	12	3	AcOH	3	75:25	0.55	-
9.0|1.00	12	4	AcOH	3	75:25	0.55	-
7.5|0.25	10	1	AcOH	3	75:25	0.55	-
7.5|0.50	10	2	AcOH	3	75:25	0.55	-
7.5|0.75	10	3	AcOH	3	75:25	0.55	25.0/39
7.5|1.00	10	4	AcOH	3	75:25	0.55	-
6.0|0.25	8	1	AcOH	3	75:25	0.55	24.2/45
6.0|0.50	8	2	AcOH	3	75:25	0.55	24.9/42
6.0|0.75	8	3	AcOH	3	75:25	0.55	23.8/39
6.0|1.00	8	4	AcOH	3	75:25	0.55	25.8/35
4.5|0.25	6	1	AcOH	3	75:25	0.55	22.7/35

4.5|0.50	6	2	AcOH	3	75:25	0.55	21.6/39
4.5|0.75	6	3	AcOH	3	75:25	0.55	22/38
4.5|1.00	6	4	AcOH	3	75:25	0.55	23.5/38
PP6.2	6	2	AcOH	3	75:25	0.55	25.3/46
PP8.2	8	2	AcOH	3	75:25	0.55	24.9/42
PP8.4	8	4	AcOH	3	75:25	0.55	-
PP10.2	10	2	AcOH	3	75:25	0.55	-
PP12.1	12	1	AcOH	3	75:25	0.55	-

Table S2: Data obtained by the rheological analysis of samples solutions described at the **Table 1**.

Sample	Consistency index – K (dyn.cm^{-2}.s)	Flow index – n (dimentionless)	Reduced Chi-Sqr	R-Square (COD)	Adj. R-Square
**CS 0.25 ->**	0.38 ± 0.07	0.63 ± 0.06	2.02	0.99	0.98
**CS 0.25 <-**	0.09 ± 0.02	1.01 ± 0.08	3.06	0.98	0.98
**CS 0.50 ->**	0.40 ± 0.05	0.70 ± 0.04	0.50	0.99	0.99
**CS 0.50 <-**	0.21 ± 0.02	0.88 ± 0.04	29.31	0.98	0.98
**CS 0.75 ->**	0.52 ± 0.03	0.74 ± 0.02	1.40	0.99	0.99
**CS 0.75 <-**	0.27 ± 0.05	0.91 ± 0.05	9.22	0.99	0.99
**CS 1.00 ->**	0.57 ± 0.03	0.83 ± 0.01	0.02	1.00	1.00
**CS 1.00 <-**	0.33 ± 0.03	0.97 ± 0.03	0.06	0.99	0.99
**PVA 4.5 ->**	2.18 ± 0.06	0.91 ± 0.01	11.27	1.00	0.99
**PVA 4.5 <-**	2.08 ± 0.14	0.94 ± 0.03	49.54	0.99	0.99
**PVA 6.0 ->**	5.36 ± 0.12	0.94 ± 0.02	0.07	1.00	0.99
**PVA 6.0 <-**	6.40 ± 1.76	0.84 ± 0.21	41.99	0.70	0.67
**PVA 7.5 ->**	16.27 ± 0.22	1.09 ± 0.03	1.55	0.99	0.99
**PVA 7.5 <-**	20.81 ± 0.35	0.82 ± 0.03	27.79	0.99	0.99
**PVA 9.0 ->**	72.52 ± 24.60	0.87 ± 0.21	15.92	0.87	0.85
**PVA 9.0 <-**	73.61 ± 43.27	0.87 ± 0.37	55.29	0.40	0.31
**PVA 4.5|CS 0.25 ->**	1.016 ± 0.02	0.99 ± 0.01	0.40	0.99	0.99
**PVA 4.5|CS 0.25 <-**	1.02 ± 0.03	0.99 ± 0.01	0.64	0.99	0.99
**PVA 4.5|CS 0.50 ->**	4.54 ± 0.15	0.99 ± 0.06	92.07	0.99	0.98
**PVA 4.5|CS 0.50 <-**	5.25 ± 0.80	0.70 ± 0.19	998.56	0.88	0.86
**PVA 4.5|CS 0.75 ->**	6.55 ± 1.06	1.09 ± 0.28	117.12	0.95	0.92
**PVA 4.5|CS 0.75 <-**	8.51 ± 2.11	0.76 ± 0.77	450.54	0.55	0.33
**PVA 4.5|CS 1.00 ->**	8.04 ± 1.08	0.92 ± 0.24	118.51	0.94	0.90
**PVA 4.5|CS 1.00 <-**	10.05 ± 2.46	0.73± 0.49	618.73	0.69	0.53
**PVA 6.0|CS 0.25 ->**	7.58 ± 0.05	1.00 ± 0.01	28.19	0.99	0.99
**PVA 6.0|CS 0.25 <-**	8.73 ± 0.13	0.77 ± 0.02	3.06	0.99	0.99
**PVA 6.0|CS 0.50 ->**	11.02 ± 0.39	0.91 ± 0.05	4.29	0.99	0.99
**PVA 6.0|CS 0.50 <-**	12.07 ± 0.46	0.79 ± 0.06	20.64	0.97	0.97
**PVA 6.0|CS 0.75 ->**	14.44 ± 0.34	0.94 ± 0.03	17.86	0.99	0.99
**PVA 6.0|CS 0.75 <-**	16.92 ± 0.42	0.81 ± 0.03	851.29	0.98	0.97
**PVA 6.0|CS 1.00 ->**	17.01 ± 0.36	1.08 ± 0.03	7.71	0.99	0.99
**PVA 6.0|CS 1.00 <-**	24.38 ± 2.21	0.70 ± 0.10	14.79	0.98	0.98

**PVA 7.5 ǀ CS 0.25 ->**	15.93 ± 0.44	1.07 ± 0.04	0.26	0.99	0.99
**PVA 7.5 ǀ CS 0.25 <-**	19.33 ± 2.82	0.87 ± 0.20	14.32	0.80	0.77
**PVA 7.5 ǀ CS 0.50 ->**	28.13 ± 1.51	0.84 ± 0.03	0.51	0.99	0.99
**PVA 7.5 ǀ CS 0.50 <-**	24.28 ± 1.52	0.90 ± 0.03	0.97	0.99	0.99
**PVA 7.5 ǀ CS 0.75 ->**	68.13 ± 4.79	0.78 ± 0.03	15.16	0.99	0.99
**PVA 7.5 ǀ CS 0.75 <-**	49.08 ± 6.75	0.91 ± 0.07	34.33	0.98	0.97
**PVA 7.5 ǀ CS 1.00 ->**	59.80 ± 2.21	0.80 ± 0.02	955.23	0.99	0.99
**PVA 7.5 ǀ CS 1.00 <-**	46.60 ± 5.00	0.91 ± 0.05	71.20	0.99	0.99
**PVA 9.0 ǀ CS 0.25 ->**	41.39 ± 1.72	0.86 ± 0.02	4.97	0.99	0.99
**PVA 9.0 ǀ CS 0.25 <-**	39.52 ± 2.74	0.87 ± 0.04	5.94	0.99	0.99
**PVA 9.0 ǀ CS 0.50 ->**	57.99 ± 3.41	0.84 ± 0.04	1.96	0.99	0.99
**PVA 9.0 ǀ CS 0.50 <-**	55.19 ± 9.98	0.86 ± 0.12	33.04	0.90	0.89
**PVA 9.0 ǀ CS 0.75 ->**	74.09 ± 1.86	0.83 ± 0.01	4.49	0.99	0.99
**PVA 9.0 ǀ CS 0.75 <-**	44.90 ± 1.65	1.06 ± 0.09	0.18	0.99	0.99
**PVA 9.0 ǀ CS 1.00 ->**	98.68 ± 4.04	0.80 ± 0.02	151.63	0.99	0.99
**PVA 9.0 ǀ CS 1.00 <-**	101.85 ± 5.44	0.78 ± 0.03	3601.34	0.99	0.99

Table S3: Qualitative analysis of fibers electrospinnability as described at the **Table 1**.

PVA content (% w/w)	Chitosan Content (% w/w)			
	**0.25**	**0.50**	**0.75**	**1.00**
**4.5**	++	+++	+	++
**6.0**	+	+++	++	+++
**7.5**	+++	++	++	++
**9.0**	+++	++	+	+

*+++: Good fiber mat formation; **: medium fiber mat; poor fiber mat formation.

Table S4: pH of the sample solutions described at the **Table 1**.

PVA content (% w/w)	Chitosan content (% w/w)							
	**0.25**		**0.50**		**0.75**		**1.00**	
	**Mean**	**Deviation**	**Mean**	**Deviation**	**Mean**	**Deviation**	**Mean**	**Deviation**
4.5	1.113	0.041	1.340	0.151	1.24	0.120	1.550	0.046
6.0	1.290	0.030	1.486	0.040	1.44	0.052	1.570	0.052
7.5	1.483	0.035	1.546	0.006	1.63	0.040	1.527	0.040
9.0	1.406	0.105	1.433	0.042	1.52	0.067	1.573	0.067

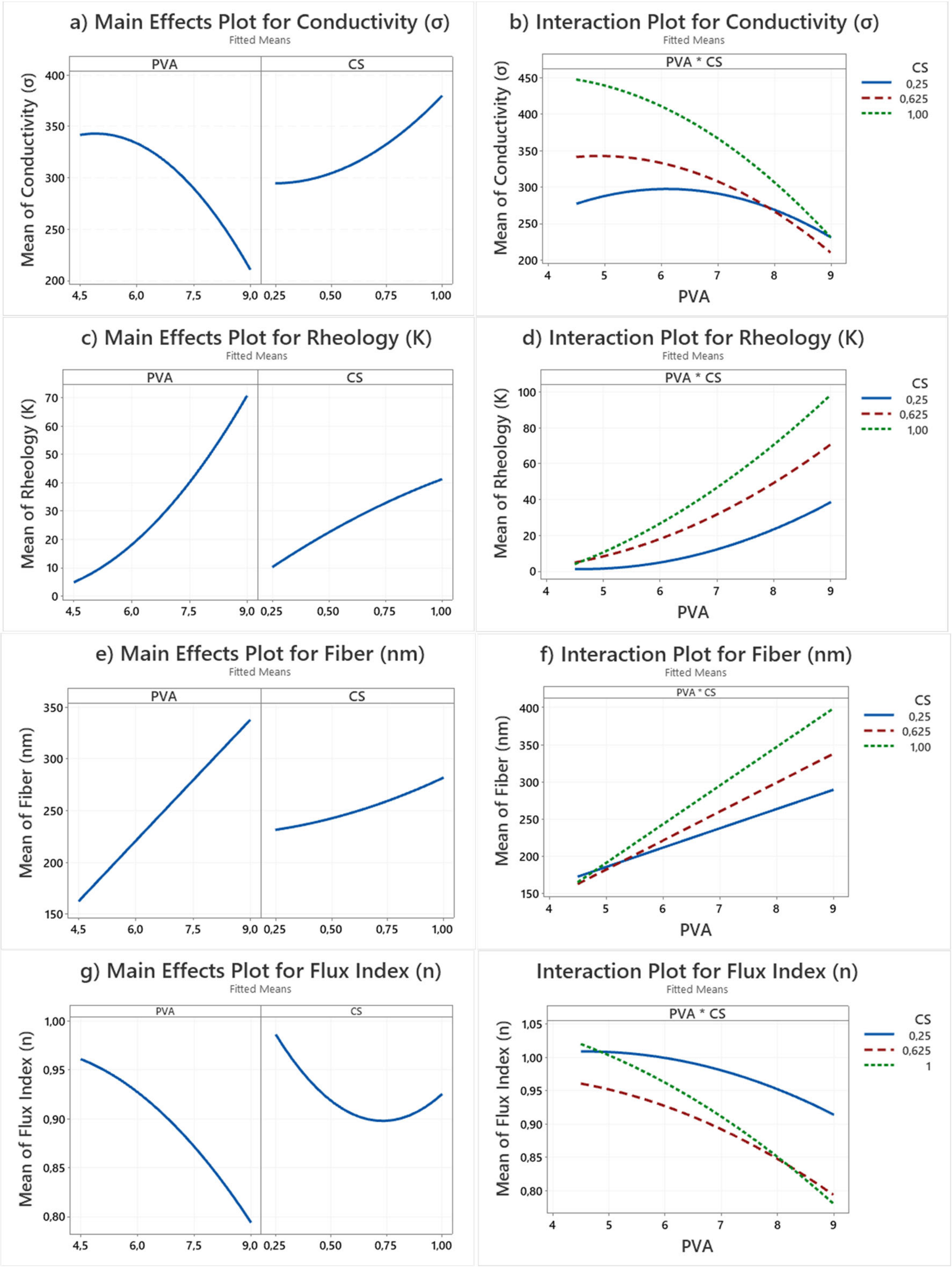

Figure S1 Main Effects and the resulting Interaction Plot for each variable, respectively, being: (**a**) and (**b**) Electrical Conductivity; (**c**) and (**d**) Rheology Consistency Index; (**e**) and (**f**) Fiber Diameter; and (**g**) and (**h**) Flow Index.
